# The Role of Coping Behavior in Healthcare Workers' Distress and Somatization During the COVID-19 Pandemic

**DOI:** 10.3389/fpsyg.2021.684618

**Published:** 2021-07-23

**Authors:** Erik Franck, Filip Haegdorens, Eva Goossens, Yannic van Gils, Michael Portzky, Francis Somville, Majed Abuawad, Stijn Slootmans, Peter Van Bogaert

**Affiliations:** ^1^Department of Nursing Science and Midwifery, Centre For Research and Innovation in Care (CRIC), Faculty of Medicine and Health Sciences, University of Antwerp, Antwerp, Belgium; ^2^Department of Public Health and Primary Care, KU Leuven, Leuven, Belgium; ^3^Research Foundation Flanders (FWO), Brussels, Belgium; ^4^Department of Patient Care, Antwerp University Hospital (UZA), Antwerp, Belgium; ^5^Department of Emergency and Traumatology, AZ St. Dimpna, Geel, Belgium; ^6^Community and Primary Health Care Centers, United Nations Relief and Works Agency, Brussels, Belgium

**Keywords:** coping, COVID-19 outbreak, healthcare workers, distress, somatization

## Abstract

**Background:** Constantly searching for a balance between work demands and their own physical and psychological health has challenged medical and nursing staff during the immediate wake of this COVID-19 viral epidemic leading to acute stress reactions and psychosomatic symptoms. Coping behavior might be a buffer for work-related stress in relation to mental well-being. The present study aims to evaluate the role of positive and negative stress-reducing activities on healthcare workers' mental and physical well-being.

**Methods:** This was a cross-sectional study using an online survey that was sent out using our network of healthcare workers at the University of Antwerp and through social media. Socio-demographic data, coping behavior with the Palliative Pallet Scale (P3), and distress and somatization using the Four-dimensional symptom checklist were collected. Surveys were completed by 1,376 participants.

**Results:** The results clearly showed that positive stress-reducing activities are related to fewer symptoms of distress and somatization. Providing direct care to COVID-19 patients was associated with a higher decrease of applying positive stress-reducing activities during the peak of the pandemic compared to the ideal situation. Finally, fewer symptoms of distress and somatization were associated with the following activities: reading, mind sports games, keeping a hobby collection, studying; engaging in sexual activities with your partner; cleaning the house, tidying up, working in the garden, doing household chores; exercising alone; walking, or taking a trip together with someone; exercise together with someone; watching TV, listening/playing (to) music/, playing computer games; playing a card game or other board game; and preparing something extra tasteful outside regular meals.

**Conclusion:** Our study demonstrated an association between concrete coping behaviors and distress and somatization in healthcare workers during the first peak of the COVID-19 pandemic. The results provide relevant and additional insights to develop and investigate interventions among others in personal leadership and resilience.

## Introduction

At the end of December 2019, some cases of severe pneumonia of an unknown etiology in Wuhan City of Hubei province were notified at the World Health Organization (WHO) subsequently termed coronavirus disease 2019 (COVID-19). The rapid global spread throughout China and across the globe led to the declaration of COVID-19 as a global health emergency (Organization, 2020). On February 4th, 2020, in Belgium the first patient was reported to have tested positive for the Coronavirus [Federal Public Service (FPS) Health, 2020]. From early March, transmission within Belgium was confirmed and the pandemic rapidly evolved with its peak of infections around the beginning of April 2020 resulting in 1,661 new infections in 1 day (Sciensano, 2020).

Hence, this was the first time that a viral outbreak at such scale occurred in Belgium. Belgian governments tried to anticipate the pandemic by prompting the reorganization of entire hospitals in a few weeks by interrupting all elective medical activities so that intensive care capacity could be enlarged to take care of COVID-19 patients. Combined with national measures to flatten the epidemic curve to prevent the healthcare system form collapsing and to reduce hospital capacity strain (Godderis et al., 2020), intensive care occupation rose to 65% of the country's total capacity for intensive care beds (Sciensano, 2020). Furthermore, in elderly care homes across Belgium, frail elderly people were infected with COVID-19, resulting in excess mortality rates in April 2020. Because healthcare workers were confronted for the first time with a viral outbreak, many of them felt not adequately skilled to provide care in such a high-risk environment. An ongoing barometer study from our department in June 2020, the aftermath of the first viral peak in Belgium, showed that 23% of the nursing workforce inadequately used protection materials and 56% overused infection control protective equipment in the care of COVID-19 patients. These results provides insights into the gaps occurring both in terms of knowledge gaps and appropriate skill set of Belgian healthcare workers (Haegdorens et al., 2021).

Moreover, studies on past viral outbreaks demonstrated the psychological impact of infectious disease outbreaks on healthcare workers working at the frontline (Khalid et al., 2016). Working in high-risk positions, completely dressed up for preventing infection, the safety risks associated with caring for patients with a highly contagious disease, and having contact with infected people that are dying without their family being at their bedside, proved to be common causes of trauma (Wu et al., 2009). Healthcare workers were forced to make unprecedented decisions on allocating scant resources to patients in need resulting in moral challenges and moral distress (Suhonen et al., 2018; Maffoni et al., 2019). Furthermore, healthcare workers were constantly searching for a balance between work demand and their physical and psychological health (Greenberg et al., 2020). The psychological sequelae observed during the SARS and EBOLA viral outbreaks indicated acute stress reactions, including psychosomatic symptoms (Tam et al., 2004; Chew et al., 2020; Xiang et al., 2020). Commonly reported symptoms ranged from physical symptoms such as pain, to fatigue, weakness, and lethargy (Leow et al., 2005). Furthermore, nervousness and anxiety experienced by staff members are most common and the intensity varied between different epidemic stages (Liao et al., 2014), but also a high prevalence of depression, insomnia, and psychological distress were reported (Lai et al., 2020).

It is clear that during this pandemic outbreak, healthcare workers were facing significant challenges in coping with the crisis. Coping is represented as actions and thoughts that individuals use to deal with challenges in their environment (Man et al., 2020). Generally, two categories of coping mechanisms are being identified: positive coping and negative coping strategies. These coping mechanisms might provide a buffering factor between work-related stressors and mental well-being. Hence, a tendency to demonstrate more positive coping behaviors when facing adversity can be considered a feature of resilience (Van Hoek et al., 2019; Nwaogu et al., 2021). However, adaptive or positive coping behaviors might also strengthen resilience. Therefore, promoting adequate coping behaviors in healthcare workers during the COVID-19 pandemic must be given priority as a highly resilient workforce is needed to face challenges over the course of this pandemic.

Also, in the first weeks of the outbreak, numerous governmental and private organizations across Flanders (Belgium) took action to mentally support frontline healthcare workers by launching websites, webinars on self-care, providing individual coaching opportunities, etc. Although the value of effective support and training is meaningful (Maunder et al., 2006), efficient and comprehensive actions should be taken in a timely fashion to protect the mental health of medical staff. However, most of these initiatives provide advice based on existing studies on coping with stress outside a pandemic outbreak and in the general population. Because every infectious disease outbreak differs in its course and no countries are alike, each has its unique impact on the healthcare staff facing that disease (Khalid et al., 2016). Furthermore, research on de impact of coping behavior, as a possible preventive factor, and the impact of specific coping behavior on the well-being during a pandemic outbreak is sparse. Consequently, the present study aims to evaluate the role of positive and negative stress-reducing activities on healthcare workers' mental and physical well-being. As such, recommendations for healthcare organizations on strategies promoting a highly resilient workforce can be formulated.

## Materials and Methods

### Study Design

This was a cross-sectional study using an online survey that was distributed *via* e-mail to our network of healthcare professionals and social media platforms such as Facebook and LinkedIn. Informed consent was provided by all participants at the beginning of the online survey. Participants were allowed to terminate the survey at any time they desired. The survey was anonymous, and confidentiality of information was assured. The American Association for Public Opinion Research (AAPOR) reporting guidelines were followed.

An online link to the survey was sent out using our network of healthcare workers at the University of Antwerp and through social media. The study included healthcare staff working in healthcare organizations across Flanders, Belgium. The data collection was performed between April 17 and 24 2020. During this period, the invitation for participants was repeated twice on social media. The questionnaire consisted of 4 parts: (1) socio-demographic data, (2) P3 palliative pallet scale, (3) 4DSQ—Distress, (4) 4DSQ—Somatization. It took about 15 min to complete the survey.

### Participants

The call to participate described the aim of the study and invited eligible participants to respond and complete the survey. Inclusion criteria were working as a health care professional in Belgium and being between 18 and 65 years old. A total of 1,657 completed surveys were received. A total of 281 participants did not meet the inclusion criteria and were excluded from the analysis.

### Instruments

#### Socio-Demographic Data

The first part consisted of several general questions, including, age, working experience, sex, marital status, children, education level, profession, place of work, and if the participant provided direct care for COVID-19 infected patients.

#### Palliative Behavior Scale

The P^3^ “Palliative Behavior” scale (Portzky, 2015) was used to measure the specific stress-reducing activities healthcare workers used. Palliative behavior can be seen as an indicator of coping style and it reflects an activity that is aimed at stress reduction. These activities can be divided into positive (e.g., walking) and destructive (e.g., smoking) activities. It is important to find a good balance between the different activities. A lot of destructive and not very positive activities can have harmful consequences (Portzky, 2015).

The P^3^ scale consisted of 18 items in terms of positive activities and 16 items in terms of destructive activities, every time with a scoring possibility from 1 (never) to 4 (very often). Participants were asked to indicate how often they had used such behavior during the past month and under ideal circumstances. The sum of all items results in a scoring range between 18 and 72 for positive stress-reducing activities and between 16 and 64 for destructive stress-reducing activities. The sub scores positive stress-reducing activities and destructive stress-reducing activities were used for the P3 scale as well as the specific behaviors in both scales. Both the test-retest reliability (correlations ranging from 0.71 to 0.81 for destructive and positive stress reducing activities, respectively) and validity (Cronbach's alpha ranging from 0.56 to 0.57 for positive and destructive stress reducing activities, respectively) were proven acceptable (Portzky, 2015). In the present sample, Cronbach's alpha ranged from 0.64 for positive stress reducing activities and 0.44 for destructive stress reducing activities. Note however that the author of the scale explains that higher internal consistencies are not expected in this kind of scale since the questionnaire inventories different coping behaviors that a person uses to cope with stress(ors). A person who reads to relax doesn't necessarily will also use exercise to cope with stress.

#### 4DSQ—Distress and 4DSQ—Somatization

The Four-dimensional symptom checklist is a Dutch self-report questionnaire designed to assess common psychological symptoms (Terluin et al., 2006). The questionnaire comprises 50 statements, which result in statements about four dimensions: distress (16 statements), anxiety (12 statements), depression (6 statements), and somatization (16 statements). Only the subscales distress and somatization were included in this study. The distress scale measures the kind of symptoms that people experience when they are “under stress” as a result of work pressure, psychosocial difficulties, or negative experiences (e.g., During the past week, did you feel easily irritated?). The somatization scale measure symptoms of somatic distress and somatoform disorder (e.g., During the past week, did you suffer from pain the abdomen or stomach area?). Each statement is answered using a 5-point Likert scale ranging from “1 = no” to “5 = very often, continuously.” The statements should be answered with how often complaints or symptoms have occurred in the recent past. Answers are then recoded in three categories: “1 = no” is scored 0, “2 = sometimes” is scored 1, and “3,4,5 = often or more” are scored 2 (Terluin et al., 2016). This results in a score ranging from 0 to 32 for both dimensions. Normative data is available indicating that a score above 10 is considered to be a moderately increased distress or somatization possibly resulting in impending dysfunction and higher than 20 as severely increased with serious tensions with great risk of dysfunction (absenteeism); in this case stress reduction is designated (Terluin et al., 2016). Cronbach's alpha in the present study were high, 0.94 and 0.87 for the distress and somatization subscale. The 4DSQ has been extensively tested for reliability and validity. Reliability is high (coefficients generally >0.80). Factorial, criterion and concurrent validity has been confirmed and it was found to be a valid self-report questionnaire to measure the most general, most common, expression of psychological problems throughout different populations (Terluin et al., 2004, 2006, 2016).

### Statistical Analysis

All analyses were done using SPSS Statistics for Mac OS version 26. Statistical significance was set at α = 0.05. Continuous variables were tested for normality using the method described by Kim (2013) and if the absolute skewness and kurtosis were ≤ 2 and ≤ 7, respectively, we assumed normality. Sum-scores were calculated of the P3 “Palliative Behavior” scale for positive stress-reducing activities and destructive stress-reducing activities. Furthermore, we calculated the difference between the P3 “Palliative Behavior” scale sum-scores of last month with those under normal circumstances. These difference scores estimate if there were more (positive) or less (negative) stress-reducing activities compared with pre-COVID circumstances. Additionally, sum-scores of the 4DSQ-distress and -somatization were calculated and cut-off points (low, medium, and highly elevated scores) described by Terluin et al. (2006, 2008) were used in further analyses. Multiple linear regression analyses were fitted using the backward elimination method to investigate the relation between the change in positive and negative behavior scores on distress and somatization scores. Finally, multiple linear regression models were fitted including each of the P3 behavior scale subitems and confounders with as a dependent variable the distress or somatization score. Holm's Sequential Bonferroni Procedure was used to correct our findings for familywise error rates and to avoid alpha inflation (Ludbrook, 1998).

### Ethical Considerations

Data were collected taking into account European legislation regarding the “General Data Protection Regulation” (=GDPR—General Data Protection Regulation). Because this concerns a study in which only adult healthcare workers participate on their own free will and after informed consent, based on the ICH-GCP principles (https://www.ema.europa.eu/en/documents/scientific-guideline/ich-e-6-r2-guideline-good-clinical-practice-step-5_en.pdf) ethical approval was not sought for the present study. Informed consent was provided by all participants at the beginning of the online survey. Participants were allowed to terminate the survey at any time they desired. The survey was anonymous, and confidentiality of information was assured.

## Results

Surveys were completed by 1,376 participants, 999 respondents (72.6%) were direct care staff (see Table 1). Almost 9% were management staff (*N* = 123), 6.7% medical doctors (*N* = 92), and 11.8% were either supportive staff or paramedics (*N* = 162).

**Table 1:** Sample characteristics in total and compared between COVID-19 and other caregivers.

	**Provided COVID-19 care**			
**Sample characteristics**	**Yes** **(*n* = 949)**	**No** **(*n* = 427)**	***p*-value**	**Total** **(*n* = 1,376)**
Age, mean (SD)	40.0 (11.5)	40.5 (10.9)	0.447	40.2 (11.3)
Working experience, mean (SD)	16.2 (11.7)	16.0 (10.9)	0.770	16.1 (11.4)
Females, % (*n*)	89.0 (845)	94.4 (403)	0.002	90.7 (1,248)
Married, % (*n*)	72.8 (691)	70.5 (301)	0.374	72.1 (992)
Has children, % (*n*)	56.4 (535)	61.1 (261)	0.099	57.8 (796)
Education level				
Undergraduate level, % (*n*)	23.4 (222)	29.3 (125)	<0.001	25.2 (347)
Bachelor level, % (*n*)	58.6 (556)	46.8 (200)		54.9 (756)
University level, % (*n*)	18.0 (171)	23.9 (102)		19.8 (273)
Profession				
Direct care (nurses, nursing aids, …), % (*n*)	78.4 (744)	59.7 (255)	<0.001	72.6 (999)
Auxiliary staff, % (*n*)	6.6 (63)	23.2 (99)		11.8 (162)
Management, % (*n*)	7.4 (70)	12.4 (53)		8.9 (123)
Physicians, % (*n*)	7.6 (72)	4.7 (20)		6.7 (92)
Place of work				
Hospital, % (*n*)	71.2 (676)	48.0 (205)	<0.001	64.0 (881)
Home care services, % (*n*)	14.8 (140)	29.3 (125)		19.3 (265)
Residential care services, % (*n*)	14.0 (133)	22.7 (97)		16.7 (230)

*Percentages calculated within columns; p-values of proportions calculated with Pearson's chi-squared test and continuous variables using an independent t-test*.

The majority of the sample is female (90.7%). The average age is 40.2 years, and the average work experience is 16.1 years. Over 70% are living together with a partner (and/or children) and 20% have no partner (with or without children). Over 70% have a college or master's degree. Most respondents work in a hospital (64%). Of these respondents, 80% work in an acute care hospital with almost one-fifth working on the emergency or intensive care unit and one-fifth on an internal medicine ward. Up to 69% comes into contact with COVID-19 infected patients.

There are significantly fewer females providing care for COVID-19 patients compared with the group providing non-COVID care (89 vs. 94%, respectively, *p* = 0.002). Healthcare workers providing COVID care were predominantly direct care professionals who had a higher education level compared with non-COVID care workers (see Table 1).

In Table 2, we compared normal (in ideal circumstances) with current (during the past month) palliative behavior scale scores for positive and destructive stress-reducing activities. The positive stress-reducing activity score was lower in the past month compared with ideal circumstances (mean diff. −3.8) and was more pronounced in healthcare workers providing direct care for COVID-patients (mean diff. −4.2). Additionally, the destructive stress-reducing activity score slightly increased in both groups when comparing the last month with ideal circumstances (mean diff. +0.3).

**Table 2:** Comparing palliative behavior scale scores for positive stress reducing activities and destructive stress reducing activities.

	**P3 positive behavior**			**P3 destructive behavior**		
	**Normal**	**Current**	**Mean difference** **(95% CI)**	**Normal**	**Current**	**Mean difference** **(95% CI)**
Total (*n* = 1,376)	42.8 (5.0)	39.0 (5.7)^*^	−3.8 (−4.2 to −3.5)	25.0 (3.0)	25.3 (3.4)^*^	0.3 (0.2–0.4)
Provided COVID-19 care (*n* = 949)	43.0 (5.1)	38.8 (5.8)^*^	−4.2 (−4.6 to −3.8)	25.1 (3.0)	25.3 (3.5)^*^	0.3 (0.1–0.5)

*Mean differences calculated between current scores and normal scores using a paired t-test; P3, palliative behavior scale.*

* *p < 0.001*.

Differences in P3 behavior scores, distress, and somatization scores between demographic variables were also investigated (Table 3). Women showed lower distress and somatization scores in general, however, no difference of change in positive or destructive behavior was found between men and women. Significantly lower distress and somatization scores were observed in married, non-COVID-care, higher educated, and home care healthcare workers. Personnel providing COVID-care showed a higher change (in the last month—ideal circumstances) in the positive behavior score compared with non-COVID workers (−4.2 vs. −3.0, *p* = 0.001) but no difference was observed in the destructive behavior score. We found that physicians, of all professions included in this study, showed the lowest distress and somatization scores. However, physicians demonstrated a slightly higher increase in destructive behavior compared with other professions. Management staff showed no change in destructive behavior, but the positive behavior score decreased greatly compared with other professions.

**Table 3:** Comparing P3 behavior scale, distress scale, and somatization scale scores between sample characteristics.

	**Change in P3 behavior scale**			
	**Δ positive** **behavior**	**Δ destructive** **behavior**	**Distress**	**Somatisation**
Men	−3.9 (6.2)	0.3 (2.4)	14.0 (8.5)	8.5 (6.2)
Women	−3.1 (5.3)	0.5 (2.0)	11.0 (7.8)	5.6 (5.5)
*p*-value	0.146	0.396	<0.001	<0.001
Married: yes	−3.7 (6.2)	0.2 (2.3)	13.3 (8.4)	8.0 (6.1)
Married: no	−4.1 (5.9)	0.5 (2.7)	14.6 (8.6)	8.9 (6.2)
*p*-value	0.339	0.104	0.013	0.024
Children: yes	−3.4 (6.3)	0.4 (2.3)	13.6 (8.4)	8.1 (6.2)
Children: no	−4.3 (5.9)	0.2 (2.5)	13.9 (8.5)	8.4 (6.1)
*p*-value	0.008	0.074	0.500	0.399
Provided COVID-19 care: yes	−4.2 (6.4)	0.3 (2.4)	14.4 (8.5)	8.8 (6.4)
Provided COVID-19 care: no	−3.0 (5.6)	0.3 (2.3)	12.2 (8.1)	6.9 (5.3)
*p*-value	0.001	0.979	<0.001	<0.001
Education level				
Undergraduate level	−4.3 (6.2)	0.2 (2.6)	15.1 (8.3)	9.8 (6.4)
Bachelor level	−4.1 (6.2)	0.2 (2.4)	13.8 (8.5)	8.3 (6.1)
University level	−2.3 (5.7)	0.6 (2.2)	11.7 (8.1)	6.2 (5.6)
*p*-value	<0.001	0.050	<0.001	<0.001
Profession				
Direct care (nurses, nursing aids, …)	−4.1 (6.1)	0.2 (2.5)	14.2 (8.5)	8.7 (6.1)
Auxiliary staff	−2.4 (6.2)	0.7 (2.2)	13.4 (7.9)	7.6 (6.2)
Management	−4.9 (6.6)	0.0 (2.3)	12.9 (8.5)	7.8 (6.7)
Physicians	−2.1 (5.7)	0.8 (1.9)	10.4 (7.8)	5.3 (4.6)
*p*-value	<0.001	0.007	<0.001	<0.001
Place of work				
Hospital	−4.0 (6.3)	0.3 (2.4)	13.7 (8.4)	8.3 (6.1)
Home care services	−3.1 (5.6)	0.4 (2.2)	13.0 (8.7)	7.3 (5.8)
Residential care services	−4.0 (6.2)	0.2 (2.5)	14.5 (8.5)	9.3 (6.8)
*p*-value	0.119	0.603	0.130	0.001

*Data presented as mean (SD); differences between two groups: independent t-tests; differences between >2 groups: oneway-ANOVA test; change in P3 behavior scale = (current – normal)*.

**Figure 1:**
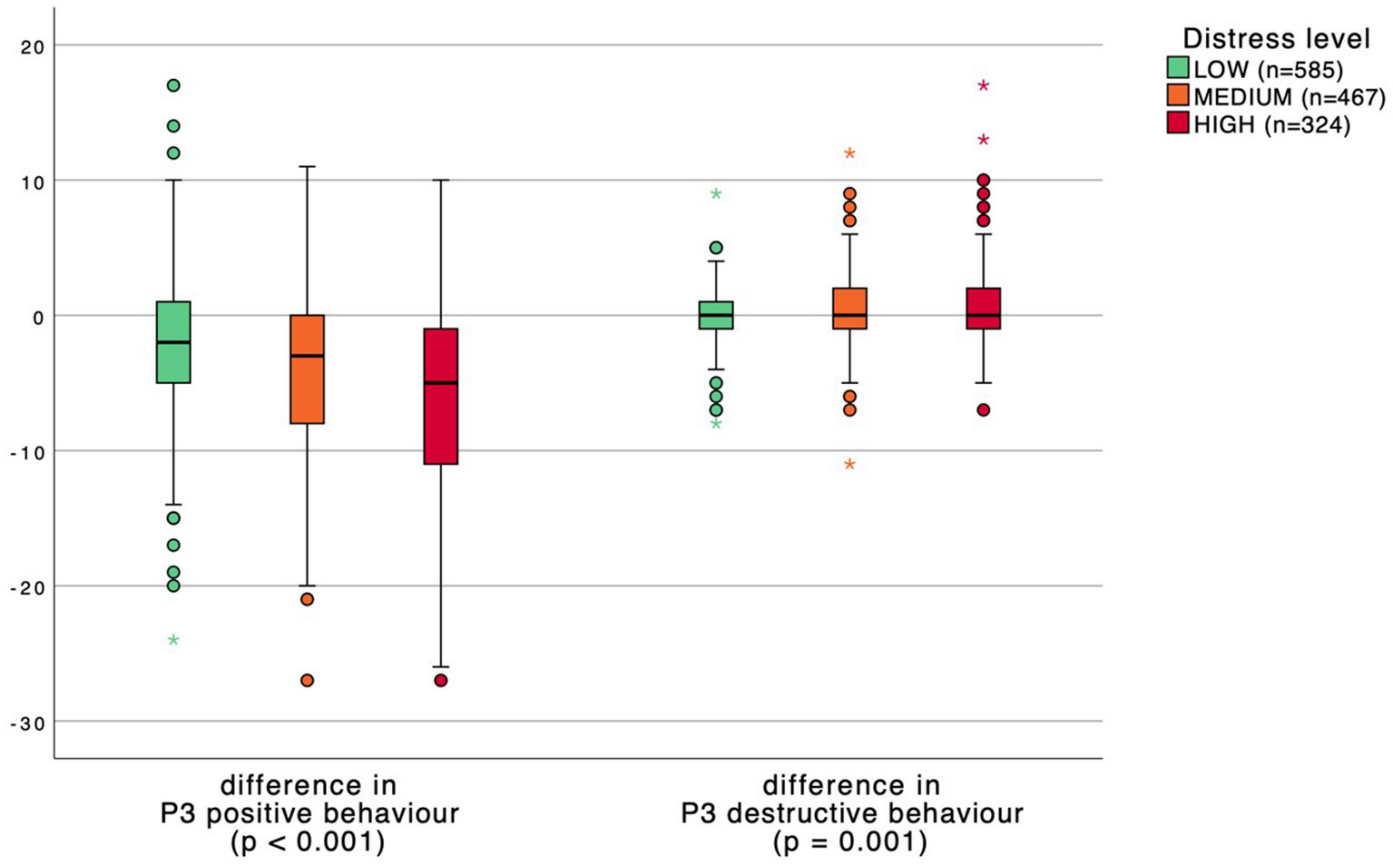
Boxplot comparing P3 behavior scores between distress levels. *p*-values calculated with a one-way ANOVA test.

**Figure 2:**
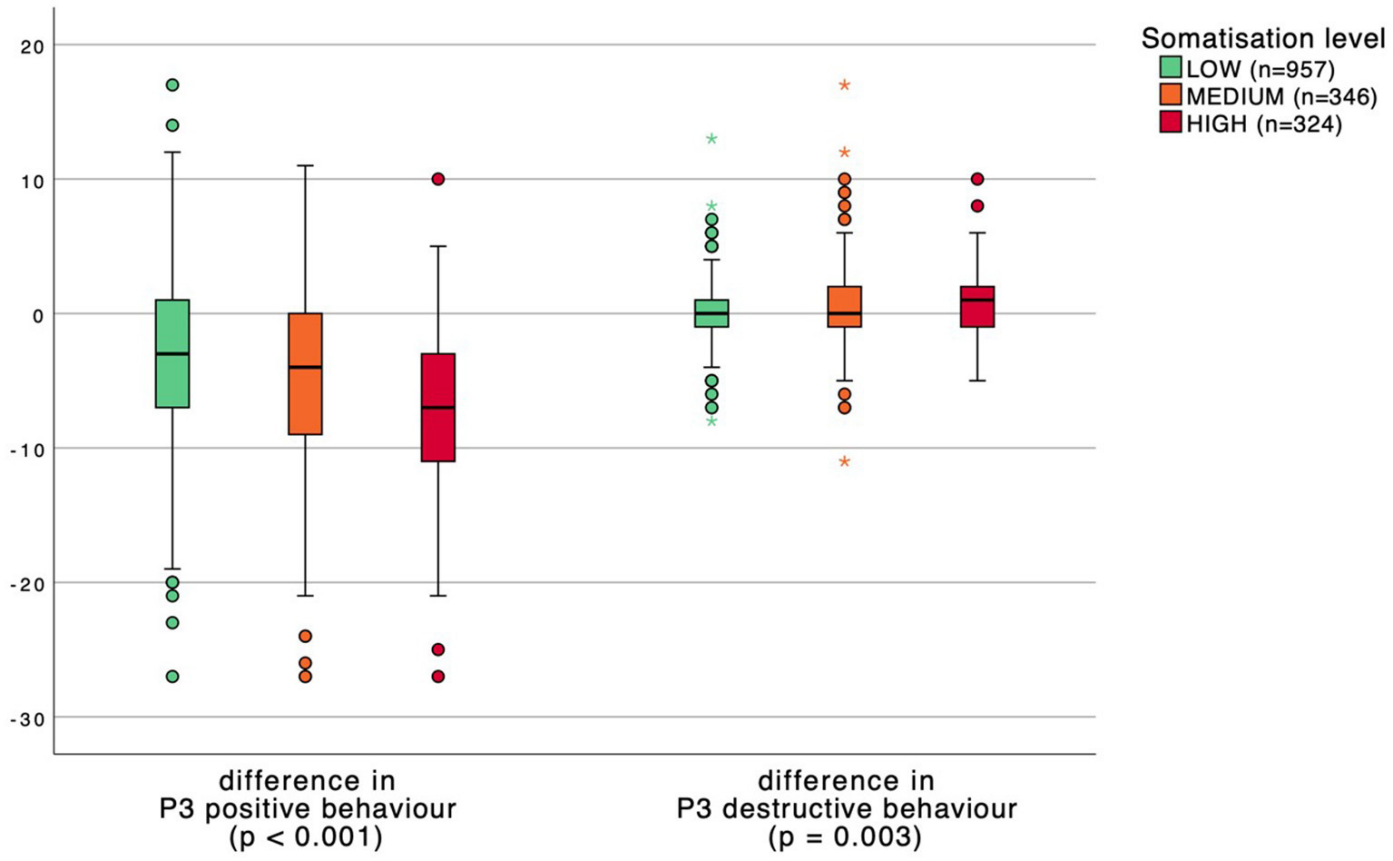
Boxplot comparing P3 behavior scores between somatization levels. *p*-values calculated with a one-way ANOVA test.

Distress and somatization levels were divided into three groups: low, medium, and high. P3 behavior scores were subsequently compared between distress and somatization levels in Figures 1, 2. A significant inverse relation was found between the change in P3 positive behavior scores and distress and somatization levels. Respondents with higher distress and somatization levels have a greater reduction in positive behavior. Moreover, healthcare workers with higher distress and somatization levels show an increase in destructive behavior scores although less pronounced.

Finally, we investigated the relation between the change in positive and negative behavior scores and distress and somatization, respectively, using a multiple linear regression analysis controlling for confounders in our dataset (Tables 4, 5). Regression models were fitted to the data using the backward elimination method. We found a negative relation that was statistically significant between positive behavior and distress and somatization controlled for other predictors in the models. Furthermore, we discovered a significant positive relation between destructive behavior and distress and somatization controlled for other predictors in the models.

**Table 4:** Multiple linear regression analysis investigating the influence of P3 behavior scores on distress.

	**Unstandardized B**	**95% CI of B**		**Standardized β**	***p*-value**
		**Lower**	**Upper**		
Change in P3 positive behavior	−0.275	−0.346	−0.203	−0.200	<0.001
Change in P3 destructive behavior	0.549	0.367	0.731	0.156	<0.001
Sex (0 = Male; 1 = Female)	2.783	1.295	4.272	0.096	<0.001
Marital status (0 = not married; 1 = married)	−1.007	−1.962	−0.051	−0.053	0.039
Providing COVID-19 care (0 = no; 1 = yes)	2.091	1.157	3.024	0.114	<0.001
Education (0 = graduate level; 1 = undergraduate level)	1.647	0.653	2.640	0.085	0.001

*n = 1,376; p-model <0.001; Variance Inflation Factor (VIF) <2; linear regression using backward elimination; adjusted R square: 0.086; excluded variables: profession: direct care, children: yes, place of work: residential*.

**Table 5:** Multiple linear regression analysis investigating the influence of P3 behavior scores on somatization.

	**Unstandardized B**	**95% CI of B**		**Standardized β**	***p*-value**
		**Lower**	**upper**		
Change in P3 positive behavior	−0.023	−0.030	−0.016	−0.179	<0.001
Change in P3 destructive behavior	0.040	0.023	0.058	0.122	<0.001
Sex (0 = Male; 1 = Female)	0.418	0.270	0.567	0.148	<0.001
Marital status (0 = not married; 1 = married)	−0.085	−0.177	0.008	−0.047	0.073
Providing COVID-19 care (0 = no; 1 = yes)	0.200	0.106	0.293	0.115	<0.001
Education (0 = graduate level; 1 = undergraduate level)	0.190	0.093	0.288	0.104	<0.001
Place of work (0 = non-residential care; 1 = residential care)	0.093	−0.007	0.193	0.051	0.070

*n = 1,376; p-model <0.001; Variance Inflation Factor (VIF) <2; linear regression using backward elimination; adjusted R square: 0.109; excluded variables: profession: direct care and children: yes; dependent variable (somatisation score) was transformed using a natural log-transformation because of heteroscedasticity*.

**Figure 3:**
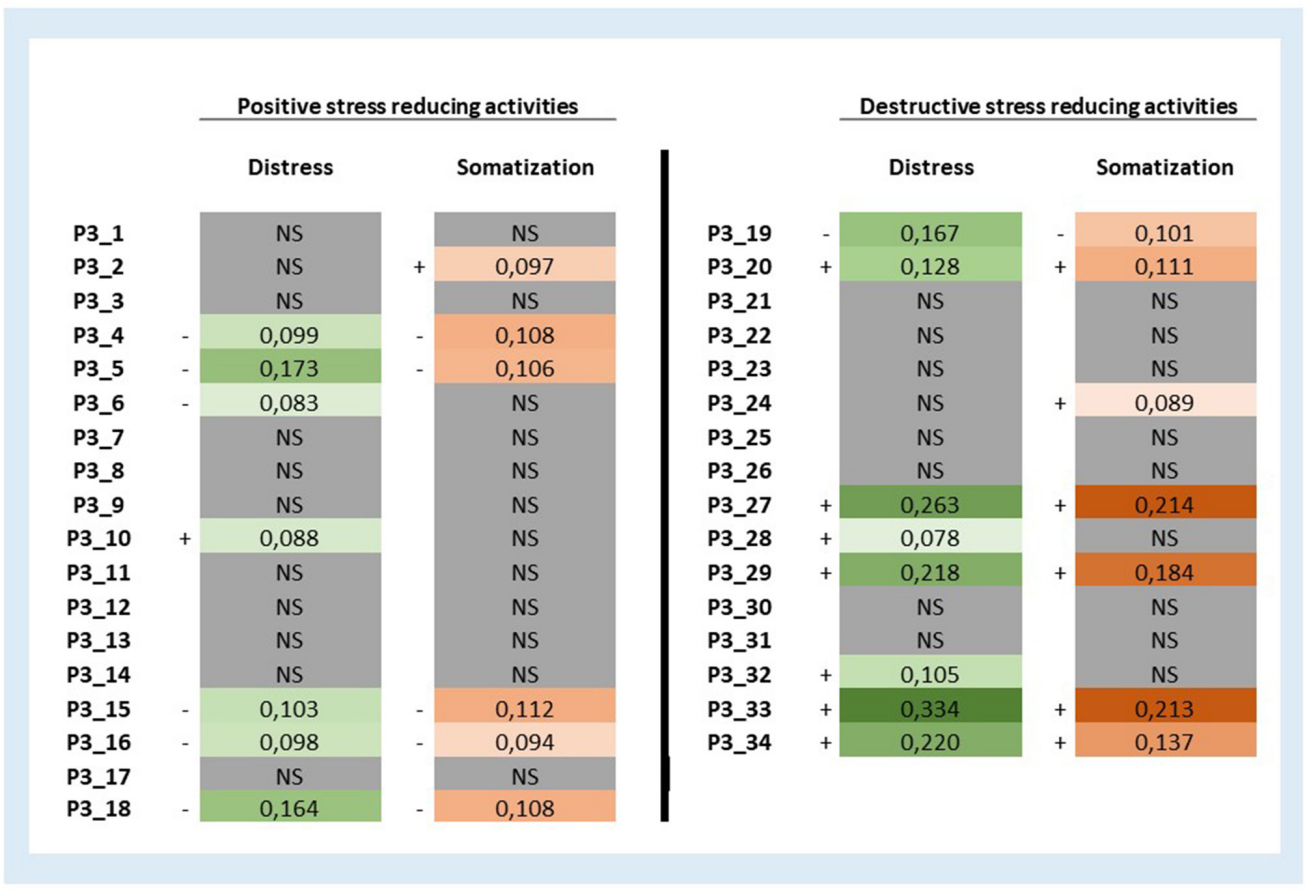
Beta coefficients of 68 multiple linear regression models investigating the relation of each positive and destructive stress-reducing activity score during the last month on distress and somatization scores. Beta correlation coefficients from 68 multiple linear regression models (34 with distress as a dependent variable and 34 with somatization as a dependent variable). Models with distress as the dependent variable were controlled for sex, married, providing covid-19 care, education: undergraduate level. Models with somatization as the dependent variable were controlled for sex, married, providing covid-19 care, education: undergraduate level, place of work: residential care. The somatization score was transformed using a natural log-transformation because of heteroscedasticity. The maximum *p*-values designated as “significant” by Holm's Sequential Bonferroni Procedure in distress and somatization models were 0.002024 and 0.000991, respectively. NS, not significant.

To investigate the *unique impact of each of the positive and destructive stress-reducing behaviors* during the last month on distress and somatizations scores we fitted 68 multiple linear regression models with each of the P3 behavior scale subitems (*n* = 68) including the same confounders from previous models (see Table 5). Results were summarized in Figure 3 in the form of beta coefficients corresponding with each of the 68 stress-reducing behaviors. We discovered that one positive stress-reducing activity showed an association with more distress (P3_10: “Check Facebook or other social media”) and one destructive stress-reducing activity showed an negative relation with distress (P3_19: “Cleaning the house, gardening, house chores”). Furthermore, we found one positive stress-reducing activity with an association with more somatization (P3_2: “Meditation, relaxation exercises, prayer, yoga”) and one destructive stress-reducing activity showed a negative relation with somatization (P3_19: “Cleaning the house, gardening, house chores”). We differentiated between activities with no, small and slightly larger association with distress and somatization and found that P3_33: “Thinking regularly about death, but without a concrete suicide plan,” P3_27: “Taking sleeping tablets, or medication to calm down,” and P3_29: “Going on strict diets, crash diets, or eating too much” had the greatest association with distress and somatization scores. Minimum and maximum explained adjusted R2 for regression models with Distress as a dependent variable are 3.6 and 14.6%, respectively. Moreover, minimum and maximum explained adjusted R2 for regression models with Somatization as a dependent variable are 6.1 and 10.6%, respectively. The correlation matrix between all study variables is provided in the Appendix.

## Discussion

The present study is unique as we questioned the coping behaviors of healthcare workers during the first peak of the COVID-19 pandemic in Belgium. Furthermore, the perception of ideal coping behaviors were investigated allowing a comparison of potential discrepancies. Moreover, we were able to relate coping behaviors with levels of distress and somatization. As predicted, the results clearly showed that positive stress-reducing activities were related to fewer symptoms of distress and somatization. Conversely, destructive stress-reducing activities were almost all related to higher levels of distress and somatization scores. Furthermore, we observed differences in coping behaviors in relation to demographic variables and profession. Overall, these results are in line with studies investigating the impact of coping behaviors on healthcare professionals during the COVID-19 pandemic from a global perspective (Ali et al., 2020; Babore et al., 2020; Lorente et al., 2020, 2021; Salopek-Žiha et al., 2020).

Čabarkapa et al. (2020) performed a rapid review on the psychological impact of the COVID-19 pandemic including ways to address it. In their article, 13 studies considered coping strategies. Differences in coping behaviors between healthcare professionals were observed. Whereas, medical doctors appeared to be more likely to use “planning” as a coping strategy, nurses and healthcare assistants were more likely to use “behavioral disengagement” and “self-distraction.” In the present study, we found significant differences in coping strategies on both positive and negative stress-reducing activities scales of the P3 between different study groups. The decrease of using positive stress-reducing activities during the peak of the pandemic compared to ideal was highest in direct care workers and managers. As for destructive behavior, the medical doctors and auxiliary staff members showed the highest increase during the pandemic peak. Furthermore, providing direct care to COVID-19 patients was associated with a higher decrease of applying positive stress-reducing activities at peak momentum as compared to the ideal situation. Overall, as frontline medical staff members experience higher emotional turmoil (Khalid et al., 2016; Cai et al., 2020) resulting from taking care of COVID-19 infected patients, it might be that healthcare workers do not always have the appropriate skills to cope with such acute stressors often leading to disengagement, avoidance, and emotional suppression. Cai et al. (2020) reported that frontline staff members believed they have the professional obligation to work long hours. Our findings are in line with existing literature suggesting that using avoidance strategies in an attempt to avoid unnecessary interactions or new COVID-19 information to escape from stressors caused by overly engaging (Eslami Akbar et al., 2015; Vagni et al., 2020; Windarwati et al., 2021).

Moreover, our study went beyond existing research as we were able to analyze the effect of each of the 34 positive or destructive stress-reducing activities used on distress and somatization scores of healthcare workers during the first peak of the COVID-19 pandemic. These analyses revealed some interesting results. Lower distress or somatization scores are associated with the following positive stress reducing activities: “reading, brain games, hobbies, modeling, studying;” “erotic or sexual activities with a partner;” “going to the gym or doing sports, by yourself;” “going for a walk or take a little trip (in group or at least with someone else);” “going to the gym or doing sports, with someone else;” and “watching TV, listening to music/playing music, playing computer games.” We discovered also one destructive stress reducing activity associated with lower distress and somatization scores (i.e., cleaning the house, tidying up, working in the garden, doing household chores). Conversely, higher symptoms of distress or somatization were associated with the following destructive stress reducing activities: “thinking regularly about death, but without a concrete suicide plan;” “Taking sleeping tablets, or medication to calm down;” “thinking about a concrete suicide plan, considering an attempt;” “going on strict diets, crash diets, or eating too much;” “eating candy or junk food;” “Self-mutilation, or other deliberate self-hurting behavior;” “using soft drugs;” and “drinking coffee/tea or caffeinated softdrinks.” Finally, we found two positive stress reducing activities associated with higher distress or somatization scores (i.e., “meditation and relaxation exercises” and “using Facebook or other social network websites”). Consequently, based on these results we can recommend health care professionals during peaks in the COVID-19 pandemic crisis to engage in concrete coping behaviors aimed at behavioral activation, keeping the daily structure and distraction. On the other hand, health care workers should be warned that coping behavior aimed at avoidance is associated with more detrimental effects on their mental and physical health. Having suicidal ideations or concrete suicidal thoughts or plans on the other hand must be considered a warning sign for immediate psychological support.

Overall, these findings are in line with general research in psychology investigating the impact of different coping strategies on mental and physical well-being. Coping was in the transactional model of Lazarus and Folkman (1987) a phenomenon to manage internal and/or external stressors that are perceived to exceed their personal resources people use both cognitive and behavioral coping responses. They identified two interdependent strategies working together identified as direct action or problem-focused coping and palliative or emotion-focused coping. In this study, lower use of positive and higher use of destructive stress-reducing behaviors was associated with more detrimental outcomes on distress and somatization. This is in line with other research demonstrating that an avoidant coping style was associated with increased levels of depression, anxiety, and loneliness (Minahan et al., 2021). Moreover, our findings are in line with the stress and coping framework of Lazarus and Folkman (Lazarus and Folkman, 1984) suggesting that the relationship between stress and psychological well-being is mediated by dysfunctional coping. Due to the correlational nature of our study, it is not possible to draw causal conclusions among the variables considered, nevertheless, we believe that our findings may contribute to understand healthcare workers' stress reactions involved in a pandemic outbreak in Flanders, Belgium.

The present study has limitations to be considered when interpreting the results. First of all, we used our own network and social media to distribute the survey. Therefore, selection bias could have occurred and no response rate can be calculated. Furthermore, the use of a cross-sectional design does not allow us to infer causality for the relationships examined. Second, because we asked participants to rate their coping behaviors at present and ideal circumstances, recall bias could have influenced the results. In the future, a longitudinal study design with a well-defined study population of healthcare workers and their work conditions is recommended. Finally, the study groups (professions) were unequal in numbers. The largest group were direct care workers. Generalization to other groups must be done with caution.

Using valid survey instruments we investigated coping behavior in relation to distress and somatization in a relevant number of healthcare workers during the course of the COVID-19 pandemic. The study results provides relevant and additional insights to develop and investigate interventions among others in personal leadership and resilience. We, therefore, recommend healthcare organizations not only monitor the mental and physical well-being of their staff members but also provide a concrete list of activities on how to cope with the psychological challenges during this COVID-19 or other pandemic outbreaks. Interventions from the field of positive psychology should be challenged.

## Conclusion

This cross-sectional study investigated the relationship of coping behavior and distress and somatization in healthcare professionals working during the COVID-19 pandemic. The results clearly showed that positive stress-reducing activities are related to fewer symptoms of distress and somatization. Providing direct care to COVID-19 patients was associated with a higher decrease of applying positive stress-reducing activities during the peak of the pandemic compared to the ideal situation. Finally, positive stress reducing activities were associated with fewer symptoms of distress and somatization. We, therefore, recommend healthcare organizations not only monitor the mental and physical well-being of their staff members but also provide interventions on how to cope with the psychological challenges during this COVID-19 or other pandemic outbreaks.

## Data Availability Statement

The datasets presented in this study can be found in online repositories. The names of the repository/repositories and accession number(s) can be found at: https://anet.uantwerpen.be/desktop/irua.

## Ethics Statement

Ethical review and approval was not required for the study on human participants in accordance with the local legislation and institutional requirements. The patients/participants provided their written informed consent to participate in this study.

## Author Contributions

EF, FH, EG, and PV contributed to the conception, design, analysis, interpretation of the data, and have drafted and reviewed the work. YG, MP, FS, MA, and SS have substantially revised the work and contributed to the data acquisition. All authors contributed to the article and approved the submitted version.

## Conflict of Interest

The authors declare that the research was conducted in the absence of any commercial or financial relationships that could be construed as a potential conflict of interest.

## Publisher's Note

All claims expressed in this article are solely those of the authors and do not necessarily represent those of their affiliated organizations, or those of the publisher, the editors and the reviewers. Any product that may be evaluated in this article, or claim that may be made by its manufacturer, is not guaranteed or endorsed by the publisher.

## Figures and Tables

**Appendix 1:**
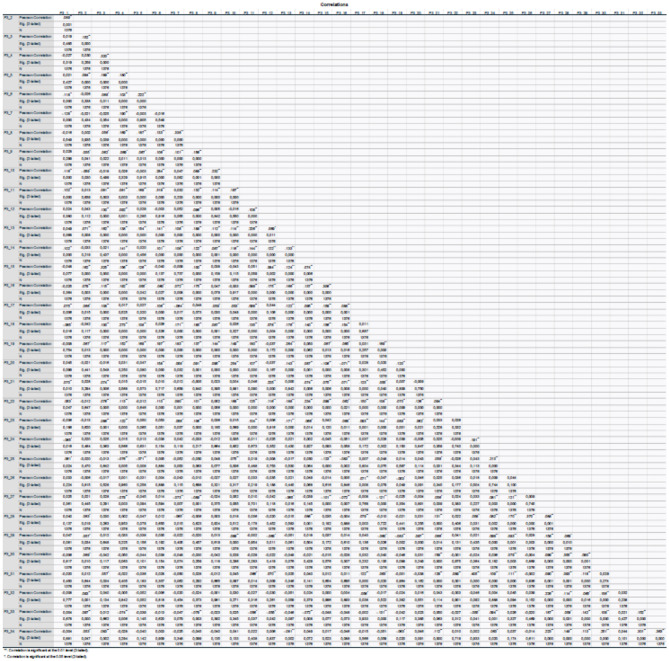
Correlation matrix between the items of the P^3^ Palliative Behavior Scale.
