# Supplementary material for: The Role of Coping Behavior in Healthcare Workers' Distress and Somatization During the COVID-19 Pandemic
Source: Front Psychol. 2021 Jul 23;12:684618. doi: 10.3389/fpsyg.2021.684618 (PMC8342849; doi:10.3389/fpsyg.2021.684618)

Relative Weights Analysis using the method described by Tonidandel & LeBreton (2015) <DOI:10.1007/s10869-014-9351-z>
Dependent variable: Somatization score

	Variables	Raw,RelWeight	Rescaled,RelWeight	Sign	Sign,Rescaled,RelWeight
27	P3_27	0,03204056	17,19757231	+	17,19757231
33	P3_33	0,030032907	16,11997704	+	16,11997704
29	P3_29	0,025895415	13,89920359	+	13,89920359
20	P3_20	0,011776837	6,321144048	+	6,321144048
1	P3_1	0,007628043	4,094305083	+	4,094305083
34	P3_34	0,007339875	3,939632263	+	3,939632263
2	P3_2	0,006377165	3,422903706	+	3,422903706
24	P3_24	0,003927102	2,107847851	+	2,107847851
22	P3_22	0,002189401	1,175147198	+	1,175147198
32	P3_32	0,001915026	1,027878251	+	1,027878251
9	P3_9	0,00149964	0,804922461	+	0,804922461
25	P3_25	0,001286051	0,690280151	+	0,690280151
28	P3_28	0,001152553	0,618625578	+	0,618625578
10	P3_10	0,001145309	0,614737592	+	0,614737592
26	P3_26	0,00070402	0,377878176	+	0,377878176
31	P3_31	0,000397852	0,213544812	+	0,213544812
21	P3_21	0,0002321	0,12457822	+	0,12457822
23	P3_23	0,000197283	0,105890255	+	0,105890255
11	P3_11	0,000255896	0,13735039	-	-0,13735039
30	P3_30	0,000412088	0,221185415	-	-0,221185415
17	P3_17	0,000591629	0,317553422	-	-0,317553422
3	P3_3	0,000873057	0,468607702	-	-0,468607702
7	P3_7	0,000972544	0,522006818	-	-0,522006818
8	P3_8	0,001314194	0,705385737	-	-0,705385737
16	P3_16	0,002195424	1,178380445	-	-1,178380445
13	P3_13	0,002451751	1,315962097	-	-1,315962097
14	P3_14	0,002699877	1,449142403	-	-1,449142403
12	P3_12	0,00305905	1,641926367	-	-1,641926367
6	P3_6	0,003286854	1,764198554	-	-1,764198554
5	P3_5	0,004281362	2,297994511	-	-2,297994511
19	P3_19	0,004982762	2,674466528	-	-2,674466528
18	P3_18	0,00509386	2,734097852	-	-2,734097852
4	P3_4	0,00664114	3,564590727	-	-3,564590727
15	P3_15	0,011459997	6,151082446	-	-6,151082446

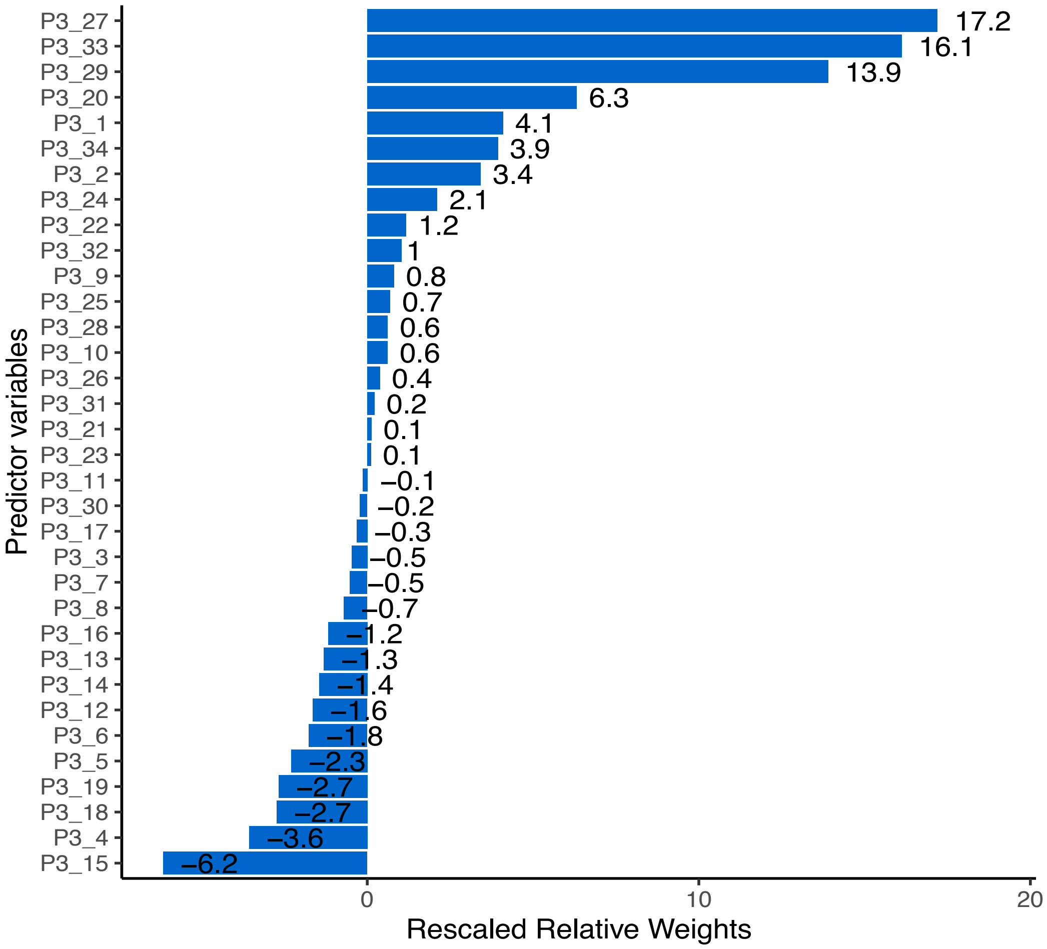

Supplement: Supplementary file 1 [file Data_Sheet_1.PDF]
