# Supplementary material for: The Role of Coping Behavior in Healthcare Workers' Distress and Somatization During the COVID-19 Pandemic
Source: Front Psychol. 2021 Jul 23;12:684618. doi: 10.3389/fpsyg.2021.684618 (PMC8342849; doi:10.3389/fpsyg.2021.684618)

Relative Weights Analysis using the method described by Tonidandel & LeBreton (2015) <DOI:10.1007/s10869-014-9351-z>
Dependent variable: Distress score

	Variables	Raw,RelWeight	Rescaled,RelWeight	Sign	Sign,Rescaled,RelWeight
33	P3_33	0,078342029	27,15962745	+	27,15962745
27	P3_27	0,043368548	15,03501513	+	15,03501513
29	P3_29	0,029300085	10,15775816	+	10,15775816
34	P3_34	0,020715013	7,181484168	+	7,181484168
20	P3_20	0,012461327	4,32009472	+	4,32009472
10	P3_10	0,007649031	2,651767403	+	2,651767403
9	P3_9	0,004589973	1,591252746	+	1,591252746
1	P3_1	0,004442824	1,540238819	+	1,540238819
32	P3_32	0,002749677	0,953258427	+	0,953258427
17	P3_17	0,001739895	0,603186913	+	0,603186913
23	P3_23	0,001600264	0,554779616	+	0,554779616
28	P3_28	0,001355928	0,470073178	+	0,470073178
26	P3_26	0,001195596	0,414489267	+	0,414489267
2	P3_2	0,000877661	0,304267679	+	0,304267679
31	P3_31	0,000705017	0,244415285	+	0,244415285
24	P3_24	0,000662253	0,229589974	+	0,229589974
25	P3_25	0,000630748	0,218667913	+	0,218667913
21	P3_21	0,000578959	0,200713769	+	0,200713769
11	P3_11	0,000524588	0,181864234	+	0,181864234
7	P3_7	0,00037189	0,128927051	+	0,128927051
22	P3_22	0,000330952	0,114734484	+	0,114734484
14	P3_14	0,000405779	0,14067549	-	-0,14067549
3	P3_3	0,000538956	0,186845333	-	-0,186845333
30	P3_30	0,000574122	0,199036805	-	-0,199036805
13	P3_13	0,001293143	0,448306976	-	-0,448306976
8	P3_8	0,001556108	0,539471786	-	-0,539471786
12	P3_12	0,002428414	0,841883133	-	-0,841883133
16	P3_16	0,002954087	1,024123321	-	-1,024123321
4	P3_4	0,004054252	1,40552887	-	-1,40552887
15	P3_15	0,006154003	2,133470863	-	-2,133470863
6	P3_6	0,006716246	2,328389343	-	-2,328389343
18	P3_18	0,012909538	4,475480875	-	-4,475480875
5	P3_5	0,01547621	5,365294913	-	-5,365294913
19	P3_19	0,019197193	6,655285902	-	-6,655285902

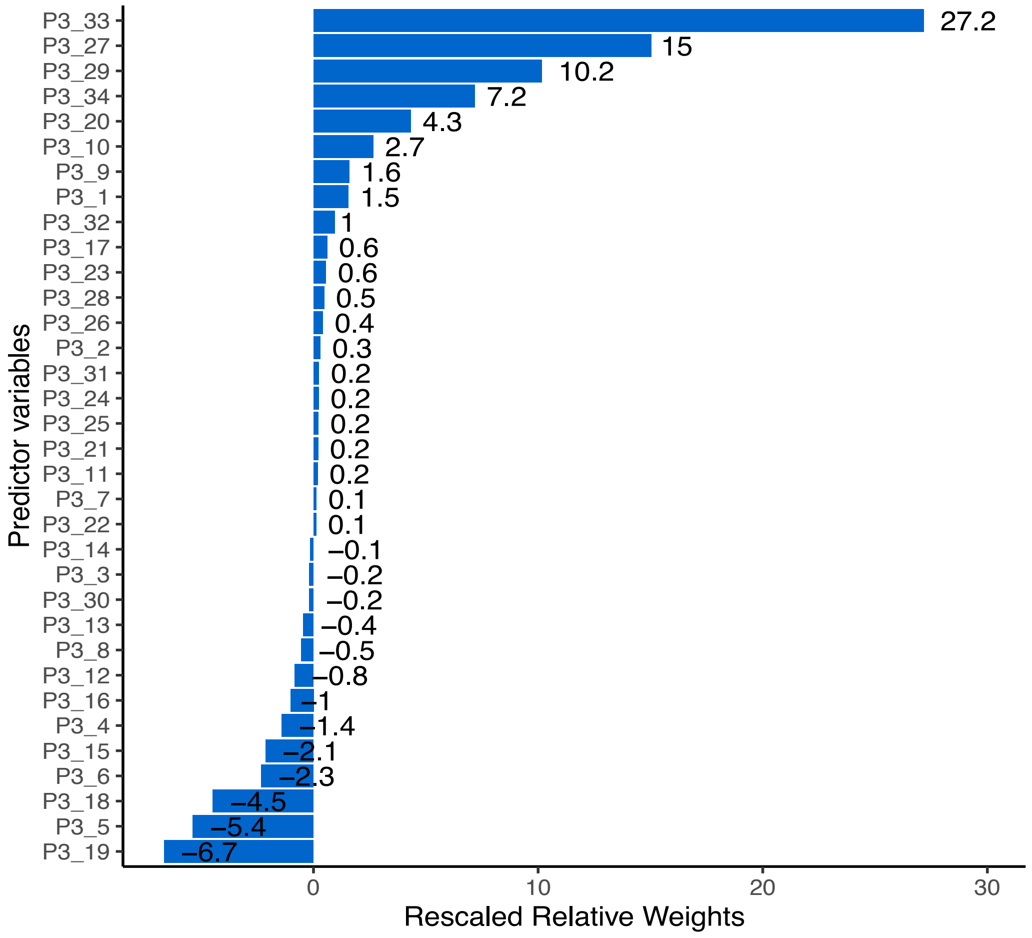

Supplement: Supplementary file 2 [file Data_Sheet_2.PDF]
